# Correction: Kowaluk et al. Physical Activity Level and Quality of Life of Children Treated for Malignancy, Depending on Their Place of Residence: Poland vs. the Czech Republic: An Observational Study. *Cancers* 2023, *15*, 4695

**DOI:** 10.3390/cancers17030440

**Published:** 2025-01-28

**Authors:** Aleksandra Kowaluk, Katarzyna Siewierska, Marie Choniawkova, Petr Sedlacek, Krzysztof Kałwak, Iwona Malicka

**Affiliations:** 1 Department of Physiotherapy, Wroclaw University of Health and Sport Sciences, 51-612 Wroclaw, Poland; katarzyna.siewierska@awf.wroc.pl (K.S.); iwona.malicka@awf.wroc.pl (I.M.); 2 Supraregional Center of Paediatric Oncology “Cape of Hope”, Wroclaw University Clinical Hospital, 50-556 Wroclaw, Poland; 3 Department of Paediatric Haematology and Oncology, University Hospital Motol, 150 06 Prague, Czech Republicpetr.sedlacek@fnmotol.cz (P.S.); 4 Department of Pediatric Hematology, Oncology and BMT, Wroclaw Medical University, 50-556 Wroclaw, Poland; krzysztof.kalwak@gmail.com

**Additional Affiliation**

In the published publication [1], there was an error regarding the affiliation for Krzysztof Kałwak. The correct affiliation should be: 4 Department of Pediatric Hematology, Oncology and BMT, Wroclaw Medical University, 50-556 Wroclaw. The authors apologize for any inconvenience caused and state that the scientific conclusions are unaffected. This correction was approved by the Academic Editor. The original publication has also been updated [1].
